# Dispersal of allergenic pollen from *Cryptomeria japonica* and *Chamaecyparis obtusa*: characteristic annual fluctuation patterns caused by intermittent phase synchronisations

**DOI:** 10.1038/s41598-019-47870-6

**Published:** 2019-08-07

**Authors:** Akira Ishibashi, Kenshi Sakai

**Affiliations:** 1 Department of Environment Conservation, Graduate School of Agriculture,Tokyo University of Agriculture and Technology, Tokyo, 183-8509 Japan; 2 Department of Environmental and Agricultural Engineering, Institute of Agriculture, Tokyo University of Agriculture and Technology, Tokyo, 183-8509 Japan

**Keywords:** Ecological modelling, Applied mathematics

## Abstract

Trees produce pollen during specific times of the year. Pollen can induce pollinosis, a type of allergic rhinitis, in humans. In Japan, allergenic pollen is mainly dispersed from February to May. Using data collected at 120 observation sites managed by the Japanese Ministry of the Environment, we studied the annual patterns of airborne allergenic pollen. The allergenic pollen showed an alternating ON–OFF cycle, but the length of the cycle differed among regions. We used an in-phase/out-of-phase analysis to quantify two characteristic features of the synchronisation. The degrees of phase synchronisation were strong in eastern and weak in western Japan. The pattern of allergenic pollen dispersal throughout Japan is typical intermittent synchronisation. This is the first study to evaluate allergenic pollen’s distribution from a phase synchronisation viewpoint.

## Introduction

Trees are important organisms that provide many benefits to humans. Natural and plantation forests provide materials, such as wood for construction and fuel, are important components of water cycles, and prevent landslides and soil loss. However, the pollen produced at certain stages of the reproductive cycles of some trees can cause pollinosis, a type of allergic rhinitis in humans. Pollinosis is triggered when the immune system overreacts to pollen that enters the body through breathing. It is often harmful to human health and causes runny noses, itchy eyes, sneezing, and fever. Some people can even develop respiratory disorders if the pollinosis becomes severe.

Many types of pollen have been identified as allergens, and people worldwide suffer from pollinosis. For instance, portions of the population react to birch pollen in central and northern Europe, olive pollen in Mediterranean areas, and ragweed pollen in Hungary^1^. Approximately 15% of the European population suffers from seasonal allergic rhinitis caused by aeroallergens, which include airborne pollen^2^. A survey using an allergic skin test showed that 10% of the American population reacts to both ryegrass and ragweed pollen^3^.

In Japan, *Cryptomeria japonica* (sugi; Japanese cedar) and *Chamaecyparis obtusa* (hinoki; Japanese cypress) are the largest and the second largest tree species, respectively, that produce allergenic pollen. Their pollen dispersal season is mainly from February to May, resulting in pollinosis being most severe during this period in Japan. It has been estimated that approximately 17% of the Japanese population (or more than 20 million people) develop pollinosis by breathing sugi pollen^4^. Sugi is economically valuable for the Japanese forest industry and is distributed almost nationwide. In the period of reconstruction in the 1950s after World War II, the domestic demand for wood for house construction increased markedly. Consequently, the Japanese government carried out large-scale afforestation to supply the market with wood. In addition, sugi trees usually produce large amounts of pollen after they reach 30 years of age. Therefore, a large amount of sugi pollen is now dispersed extensively in Japan.

The amounts of flowers or fruits vary from year to year in forest trees such as beech^5^ and oak^6^. Moreover, some forest trees show masting, the phenomenon in which the production of reproductive organs is synchronised within a population^7^. In Finland, the annual allergenic pollen distributions can be synchronised among various species (including *Betula*, *Alnus*, *Corylus*, *Salix*, and *Populus*) at several locations^8^. The degree of spatial synchronisation of annual pollen production has been studied^9^.

Previous studies on sugi pollen in Japan have mainly focused on its biological characteristics and how to predict the total annual pollen amount. In terms of biological characteristics, the mean temperature in July of the previous year is positively correlated with the amount of male flowers^10^, and a high mean temperature in summer promotes their differentiation. Therefore, the weather conditions in summer and an index of the amount of male flowers have been used to estimate the amount of airborne pollen^11–13^. It is also empirically known that the dispersal rhythms of sugi and hinoki pollen have alternate cycles of ON- (abundant amount) and OFF- (small amount) years. Conventionally, this alternate dispersal rhythm has been incorporated as a dummy variable in the regression analysis of the total pollen count^14^. In this paper, we assumed that the alternate dispersal rhythm correlated with nonlinear dynamics. Although many researchers have studied the pollen of these trees, almost all the studies have been conducted using limited nearby sites. Therefore, to date, it is unclear how allergenic pollen production is spatially synchronised in Japan. Beginning in 2003, the Japanese Ministry of the Environment launched the pollen observation system “Hanako-san”, which is available for public use. This system has recorded the dispersal of sugi pollen each year at 120 sites located throughout Japan. In this paper, we used the data supplied by this system to investigate the spatial distribution of synchronisations. The overall aim of this study was to obtain new fundamental knowledge regarding the dispersal of allergenic pollen (such as sugi and hinoki) in terms of phase synchronisation. In particular, we employed an in-phase and out-of-phase analysis to quantify the characteristic features of nationwide synchronisations of allergenic pollen. The results obtained here will be useful for the accurate prediction of airborne allergenic pollen levels. Numerical forecasts of allergenic pollen abundance are essential for both allergic individuals and clinicians^14^. Earlier predictions of nationwide pollen distribution patterns may contribute to the more effective prevention of pollinosis.

### Pollen observation system “Hanako-san”

**Figure 1:**
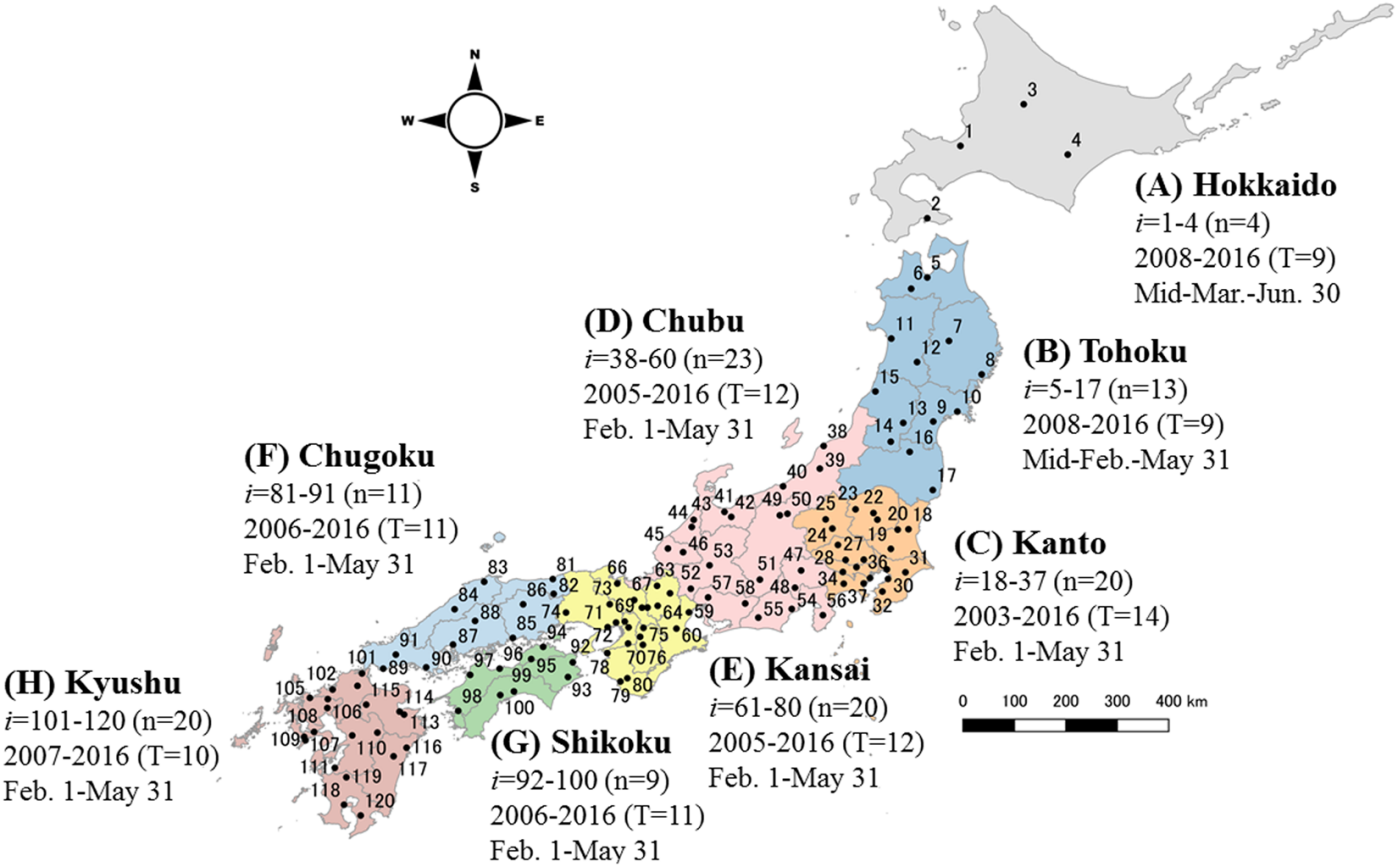
Locations of 120 observation sites for data collection. These sites were located in eight regions, covering 1,400 km north-south and 1,200 km east-west in the Japanese islands, excluding Okinawa prefecture. The longest distance among the 120 sites is 1,613 km between site 3 (lat. 43.807783, lon. 142.439397) and site 120 (lat. 43.807783, lon. 142.439397). The shape file for the map was downloaded from Geospatial Information Authority of Japan (https://www.gsi.go.jp/kankyochiri/gm_jpn.html#gm_jpn_dl) and processed with QGIS 2.18^31^.

The pollen observation system “Hanako-san” is managed by the Japanese Ministry of the Environment^15^. The data are collected at 120 observation sites located in all the Japanese prefectures, except for Okinawa. Figure 1 illustrates the locations of the 120 sites, and the annual measurement times (months) and measurement periods (years) in eight regions: (A) Hokkaido, (B) Tohoku, (C) Kanto, (D) Chubu, (E) Kansai, (F) Chugoku, (G) Shikoku, and (H) Kyushu. The number of pollen particles detected in 60 min is recorded per hour for 24 h a day during the measurement period. When the effects of snowfall and yellow sand are significant, they are excluded as anomalies from the data set. Here, *x*_*i*_ (*t*) denotes the yearly average of site *i* in year *t*.

The “Hanako-san” system employs KH-3000 (Yamatronics, Japan) automatic pollen monitors and locates them at all the observation sites. There are two types of airborne pollen measurement methods, the Duraham (gravitational) and the Burkard (volumetric). KH-3000 was designed based on the volumetric method; therefore, it can measure hourly variations in airborne pollen concentration for 24 h a day. Kawashima *et al*. described the principle and algorithm of the KH-3000 in detail^16^. They also conducted validations by comparisons with the Burkard (Hirst-type) pollen sampler and revealed high correlation coefficients between the two methods.

The accuracy verification of KH-3000 has continued to be carried out nationwide from before the system was operational in 2003 in the Kanto area until present day. The Duraham method has been mainly adopted as a reference method^17–24^. After beginning in 2003 in Kanto, operations subsequently began in 2004 in Kansai, in 2005 in Chubu, in 2006 in Chugoku and Shikoku, in 2007 in Kyushu, and in 2008 in Tohoku and Hokkaido.

In addition to sugi, the atmosphere absorbed by automatic measurement equipment contains another important tree, hinoki (*C. obtusa*). The Japanese Forest Agency reported that the sugi forest area was 4.5 million hectares, while the hinoki forest area was 2.6 million hectares in 2012. Hinoki pollen is also allergenic. The sugi forest area is 1.7 times greater than that of hinoki forests in Japan. However, the measured values do not always reflect only sugi and hinoki pollen because the measured values are based on a particle size of approximately 30 µm in diameter. Therefore, we set the period as February1^st^ to April 30^th^ for the analysis. Consequently, the data reflected mainly sugi and then hinoki pollen, and minimised the effects of other plant species, such as rice.

## Results and Discussion

### Patterns of annual variation

**Figure 2:**
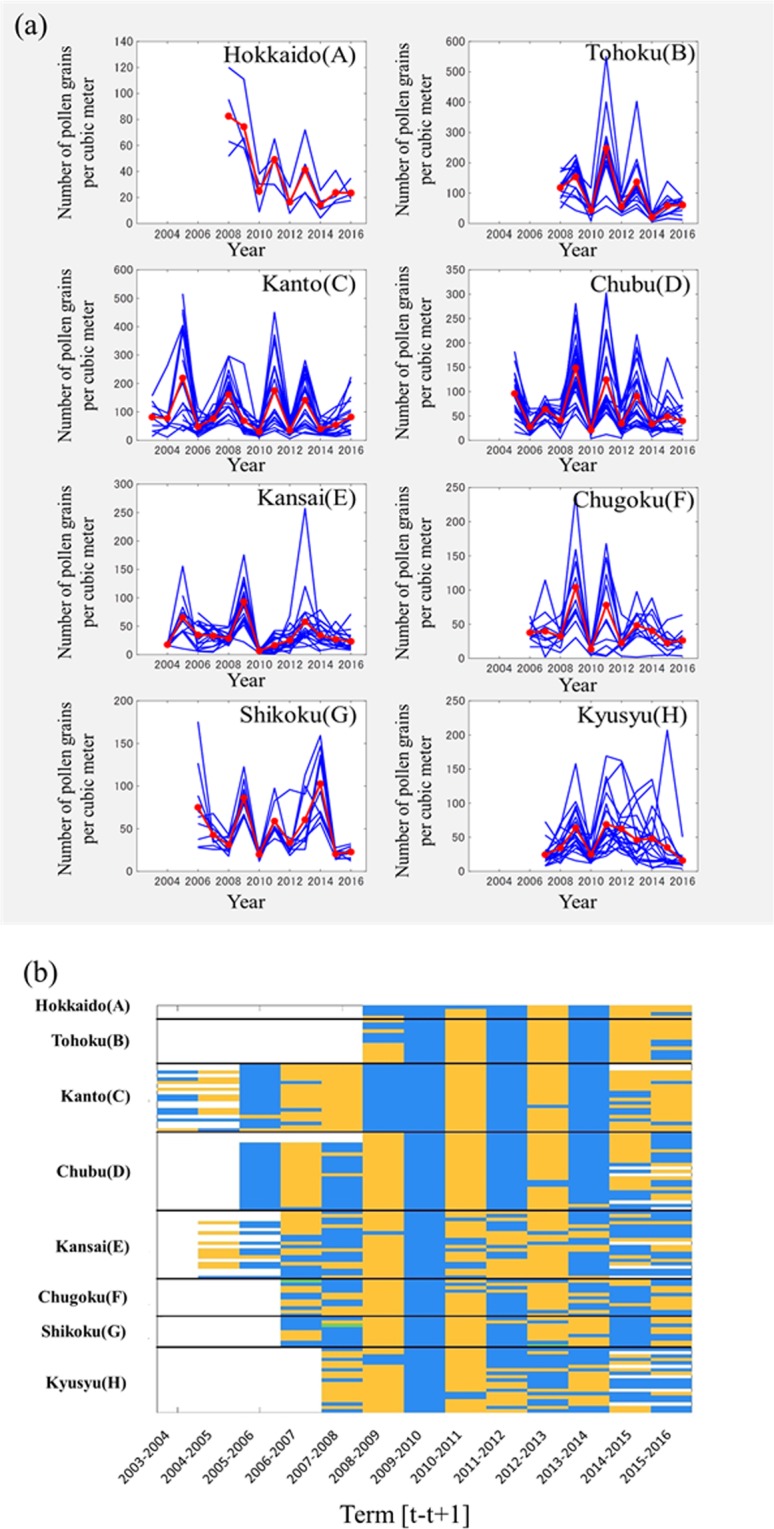
Annual airborne pollen counts *x*_*i*_(*t*) for 120 sites from 2003 to 2016. (**a**) The time series expressions of *x*_*i*_(*t*) for 120 sites in eight regions. Red lines indicate the mean value in each region. (**b**) Time–space diagrams of *x*_*i*_(*t*) in each term [*t* − *t* + 1]. Orange: increased state, Blue: decreased state, Green: unchanged state, White: no data.

Figure 2(a) shows the annual variations in the amounts of airborne pollen in eight regions [(A)–(H)]. In Tohoku and Chubu, the ON- and OFF-years alternated. The alternating ON- and OFF-year pattern in Hokkaido was similar to those in Tohoku and Chubu. In Kanto, the ON- and OFF-years alternated from 2010 to 2014. In Kansai, Chugoku, Shikoku, and Kyushu regions, located in western Japan, alternating ON- and OFF-year cycles occurred in short terms, but not over the long term as in Tohoku and Chubu.

To see the collective behaviours of 120 *x*_*i*_(*t*) more comprehensively, we used a time-space diagram as shown in Fig. 2(b). Three states are defined as below, for each term over two successive years in the [*t* − *t* + 1] term:

Increased state: $${x}_{i}({\rm{t}}+1)-{x}_{i}({\rm{t}}) > 0,$$

Decreased state: ${x}_{i}({\rm{t}}+1)-{x}_{i}({\rm{t}}) < 0$, and

Unchanged state: $${x}_{i}({\rm{t}}+1)-{x}_{i}({\rm{t}})=0$$.

In Fig. 2(b), we hypothesised that the two-year cycle is a base of the collective behaviours of 120 *x*_*i*_(*t*). However, it was clear that there are many clustering terms and sites in the western regions (E–H). Nevertheless, there are some exceptions, such as the period 2007–2008 for Kanto and the period 2015–2016 for both Kanto and Tohoku.

### Correlation analysis

**Figure 3:**
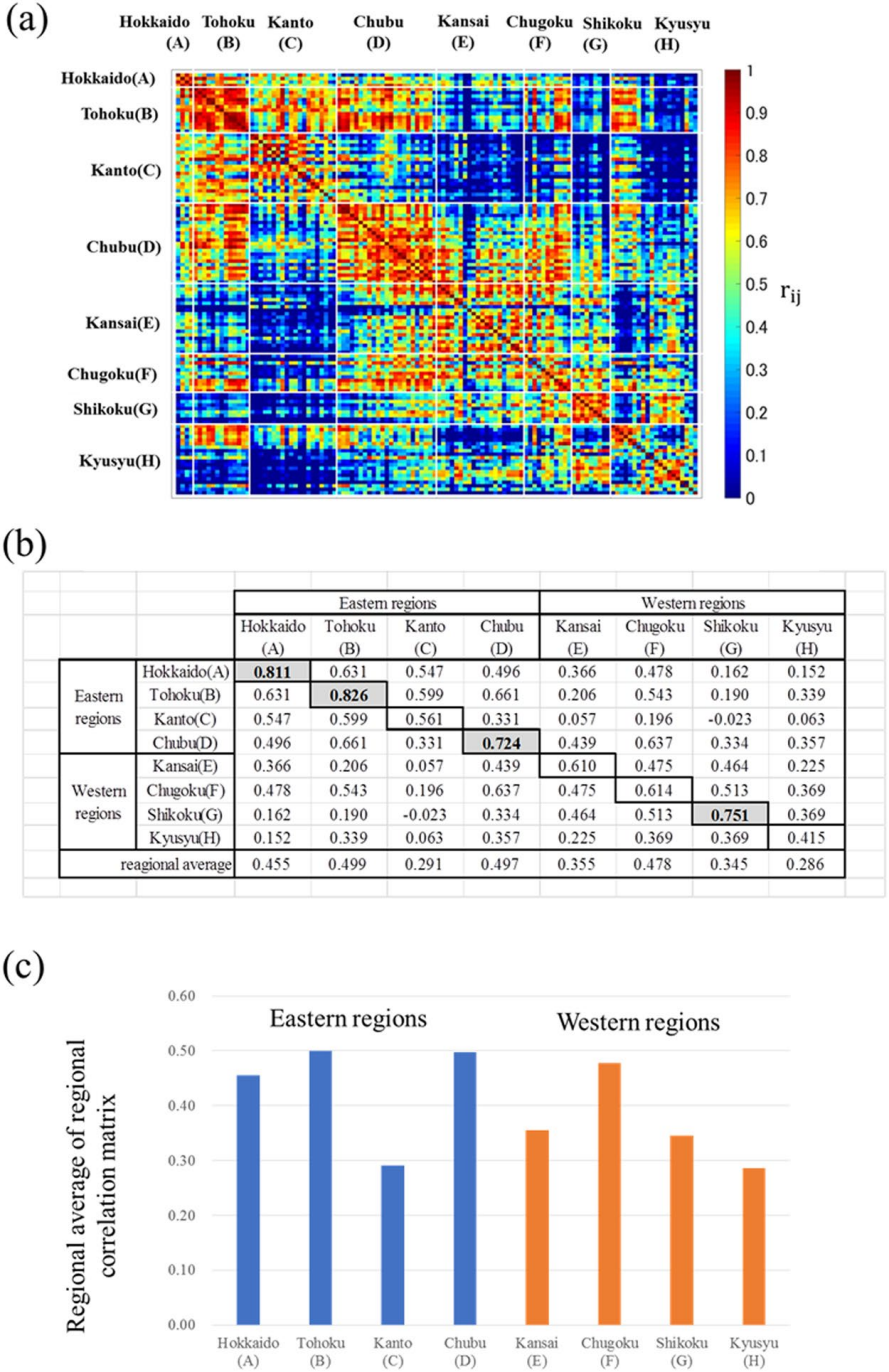
Correlation analysis for regional synchronisation. (**a**) Pearson’s correlation matrix [*r*_*ij*_]. (**b**) Regional correlation matrix [*S*_w_ + *S*_b_]. (**c**) Regional averages of the correlation matrix.

Pearson’s correlation coefficient is a conventional method to investigate synchronisation between two sites^25–27^. Here, *r*_*ij*_ denotes Pearson’s correlation coefficient between site *i* and site *j*. Figure 3(a) illustrates the correlation matrix [*r*_*ij*_] that includes all 120 observation sites. The 120 sites were arranged in the same order downwards and across the panel. The eight regions in Japan are thus represented by 64 (8 × 8) blocks surrounded by white lines.

Based on the correlation matrix, the mean of the correlation coefficients was defined, as follows, using Eq. (1) to indicate the strength of nationwide synchrony^25–27^:

1 $$S=\frac{1}{N(N-1)}\mathop{\sum }\limits_{i=1}^{N}\,\mathop{\sum }\limits_{\begin{array}{c}j=1\\ j\ne i\end{array}}^{N}\,{r}_{ij}.$$

where *r*_*ij*_ represents the Pearson’s correlation coefficient between sites *i* and *j*, and *N* represents the number of observation sites. The calculated value of *S* for the whole of Japan (i.e., all eight regions, *N* = 120) was 0.361. We defined two types of mean correlation coefficients, *S*_w_ (within a region) and *S*_b_ (between two regions), as follows for region (*l*) and region (*m*):

2 $${S}_{w}^{l}=\frac{1}{{N}_{l}({N}_{l}-1)}\,\sum _{i\in {\rm{r}}{\rm{e}}{\rm{g}}{\rm{i}}{\rm{o}}{\rm{n}}(l)}\,\sum _{\begin{array}{c}j\in {\rm{r}}{\rm{e}}{\rm{g}}{\rm{i}}{\rm{o}}{\rm{n}}(l)\\ j\ne i\end{array}}\,{r}_{ij}$$

and

3 $${S}_{b}^{l,m}=\frac{1}{{N}_{l}{N}_{m}}\,\sum _{i\in {\rm{r}}{\rm{e}}{\rm{g}}{\rm{i}}{\rm{o}}{\rm{n}}(l)}\,\sum _{j\in {\rm{r}}{\rm{e}}{\rm{g}}{\rm{i}}{\rm{o}}{\rm{n}}(m)}\,{r}_{ij}\,,$$

where *N*_*l*_ and *N*_*m*_ represent the numbers of sites in regions *l* and *m*, respectively.

The diagonal and non-diagonal components of the regional correlation matrix in Fig. 3(b) are represented by ${S}_{w}^{l}$ and $\,{S}_{b}^{l,m}$, respectively. The synchronisation of the annual fluctuation in pollen count across Japan tended to be in-phase because most *r*_*ij*_ [Fig. 3(a)], ${S}_{w}^{l}$ and ${S}_{b}^{l,m}$ [Fig. 3(b)] values are positive. The values of ${S}_{w}^{l}$ were greater than 0.7 in Hokkaido, Tohoku, Chubu, and Shikoku. However, the ${S}_{w}^{C}=0.561$ in Kanto was even less than ${S}_{w}^{E}=0.610$ in Kansai. This result is inconsistent with the behaviours exhibited in Fig. 2, in which Kanto was more synchronised than Kansai. Figure 3(c) represents the column averages of the matrices for each region; however, it is difficult to distinguish the differences between eastern and western regions.

### In-phase and out-of-phase analysis

We introduced an in-phase and out-of-phase analysis to explore phase synchronisation, because this technique is useful for two-cyclic (alternating ON–OFF) collective behaviours, as seen in Fig. 2. Prasad *et al*.^28^ expressed phase differences as in-phase and out-of-phase to quantify the extent of phase synchronisation. Fist, $\varnothing (i,j,t)$ is defined as follows;

$$\varnothing (i,j,t)=\,\{{x}_{i}(t+1)-{x}_{i}(t)\}\{{x}_{j}(t+1)-{x}_{j}({\rm{t}})\}.$$

We defined the in-phase (${f}_{{\rm{IN}}}^{{\rm{i}},{\rm{j}}}({\rm{t}})$) and out-of-phase (${f}_{{\rm{OUT}}}^{{\rm{i}},{\rm{j}}}({\rm{t}})$) fractions in Eqs (4 and 5) as follows:

4 $${f}_{{\rm{IN}}}^{{\rm{i}},{\rm{j}}}({\rm{t}})={\rm{u}}[\varnothing ({\rm{i}},{\rm{j}},{\rm{t}})]$$

and

5 $${f}_{{\rm{O}}{\rm{U}}{\rm{T}}}^{{\rm{i}},{\rm{j}}}({\rm{t}})={\rm{H}}[-\,\varnothing ({\rm{i}},{\rm{j}},{\rm{t}})].\,$$

where *i* and *j* are site locations, u[.] represents a unit step function, H[.] represents a Heaviside step function, and

The time averaged in-phase fraction between sites *i* and *j* during T years was defined as follows:

6 $${\bar{f}}_{{\rm{I}}{\rm{N}}}^{i,j}=\frac{1}{{\rm{T}}}\sum _{t}\,{f}_{{\rm{I}}{\rm{N}}}^{i,j}(t).$$

Based on the in-phase fraction, we defined *F*_IN_(*t*) as the degree of in-phase synchronisation throughout Japan for successive years, *t* and *t* + 1. *N* represents the total number of observation sites (*N* = 120). Thus:

7 $${F}_{{\rm{I}}{\rm{N}}}(t)=\frac{1}{N(N-1)}\mathop{\sum }\limits_{i=1}^{N}\,\mathop{\sum }\limits_{j=1,j\ne {\rm{i}}}^{N}\,{f}_{{\rm{I}}{\rm{N}}}^{i,j}(t)$$

The inner-regional in-phase fraction within region (*l*) was as follows:

8 $$F{{\rm{I}}{\rm{N}}}^{l}=\frac{1}{{N}_{l}({N}_{l}-1)}\sum _{i\in {\rm{r}}{\rm{e}}{\rm{g}}{\rm{i}}{\rm{o}}{\rm{n}}(l)}\,\sum _{\begin{array}{c}j\in {\rm{r}}{\rm{e}}{\rm{g}}{\rm{i}}{\rm{o}}{\rm{n}}(l)\\ j\ne i\end{array}}\,{\bar{f}}_{{\rm{I}}{\rm{N}}}^{i,j}.$$

The range of inner-regional in-phase fractions $F{{\rm{I}}{\rm{N}}}_{{\rm{w}}}^{l}$ was [0.5, 1.0], if *N* is large enough. $FI{N}_{{\rm{w}}}^{l}=1\,{\rm{a}}{\rm{n}}{\rm{d}}\,0.5$ indicated perfect in-phase and perfect out-of-phase synchronisation, respectively. The inter-regional fraction, e.g., between regions *l* and *m* was defined as follows:

9 $$F{{\rm{I}}{\rm{N}}}_{b}^{l,m}=\frac{1}{{N}_{l}{N}_{m}}\sum _{i\in {\rm{r}}{\rm{e}}{\rm{g}}{\rm{i}}{\rm{o}}{\rm{n}}(l)}\,\sum _{j\in {\rm{r}}{\rm{e}}{\rm{g}}{\rm{i}}{\rm{o}}{\rm{n}}(m)}\,{\bar{f}}_{{\rm{I}}{\rm{N}}}^{i,j}.$$

The range of inter-regional in-phase fractions $FI{N}_{b}^{l,m}$ was [0, 1.0]. $F{{\rm{I}}{\rm{N}}}_{{\rm{b}}}^{l,m}=1\,{\rm{a}}{\rm{n}}{\rm{d}}\,0$ indicated perfect in-phase and perfect out-of-phase synchronisation, respectively. For out-of-phase synchronisation,$\,{\bar{f}}_{{\rm{OUT}}}^{i,j}$, ${F}_{{\rm{O}}{\rm{U}}{\rm{T}}}(t),\,F{{\rm{O}}{\rm{U}}{\rm{T}}}_{{\rm{w}}}^{l},\,{\rm{a}}{\rm{n}}{\rm{d}}\,F{{\rm{O}}{\rm{U}}{\rm{T}}}_{b}^{l,m}$ were defined in the same ways using Eqs (5–9) respectively.

**Figure 4:**
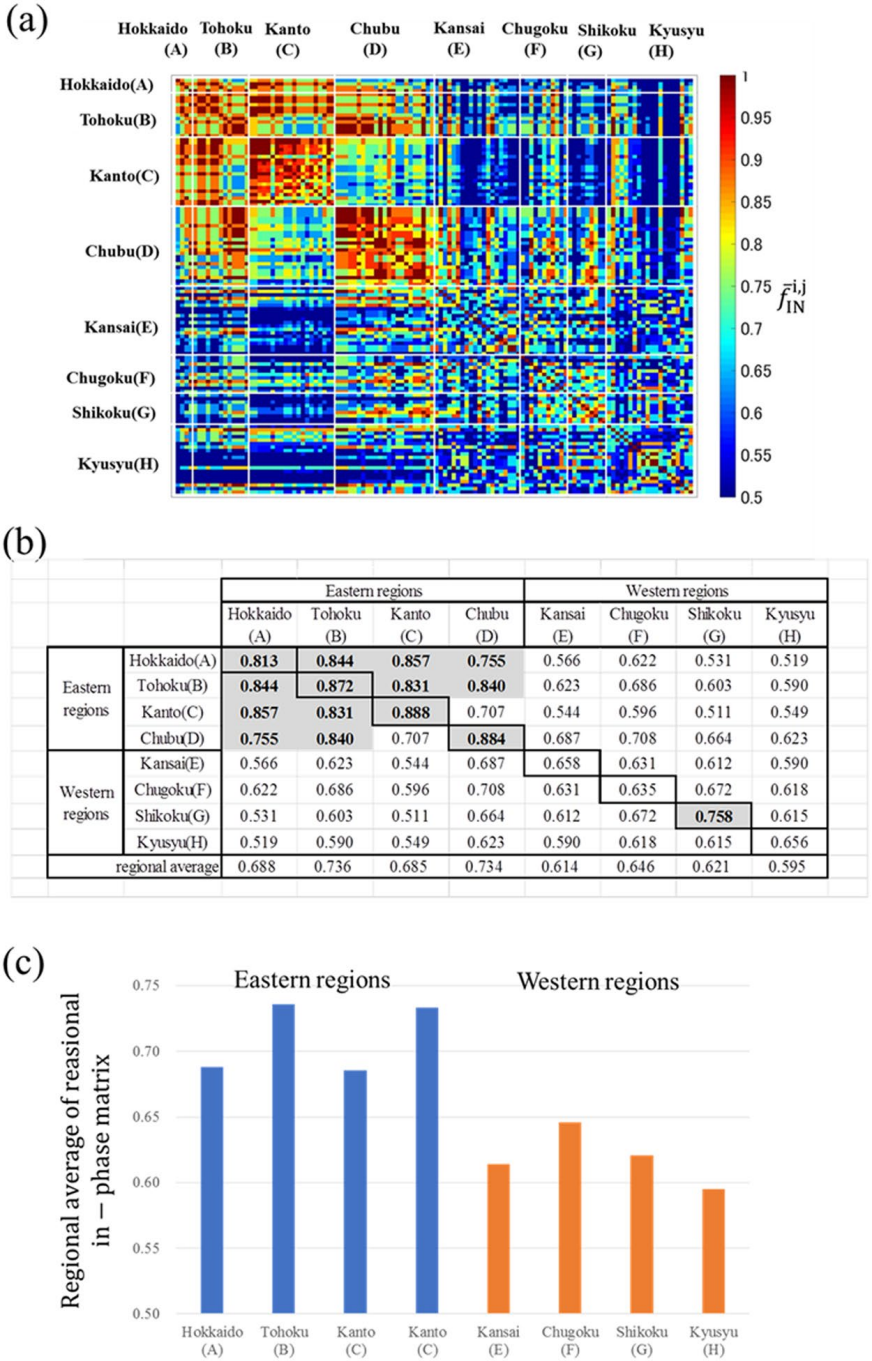
Phase analysis of regional synchronisations. (**a**) In-phase matrix $[{\bar{f}}_{{\rm{I}}{\rm{N}}}^{i,j}]$. (**b**) Regional in-phase matrix $[F{{\rm{I}}{\rm{N}}}_{w}^{l}+F{{\rm{I}}{\rm{N}}}_{b}^{l,m}]$. (**c**) Regional averages of the in-phase matrix.

To show the state of in-phase synchronisation for the 14-year measurement period, we showed the matrix $[{\bar{f}}_{{\rm{IN}}}^{i,j}]$ in Fig. 4(a). Inner-regional synchronisations were strong in eastern regions, such as Hokkaido, Tohoku, Kanto, and Chubu. Tohoku and Chubu also showed significant inter-regional synchronisations. In contrast, the degrees of synchronisation in the western regions were weaker than in the eastern regions. Figure 4(b) shows regional synchronisations with $F{{\rm{I}}{\rm{N}}}_{w}^{l}$ as diagonal components and $F{{\rm{I}}{\rm{N}}}_{b}^{l,m}$ as non-diagonal components for the eight regions and all 28 combinations. The inner-regional in-phase synchronisations (represented by the diagonal components) were very high in the four eastern regions (A–D), with Kanto showing the highest degree ($F{{\rm{I}}{\rm{N}}}_{w}^{C}$ = 0.888). This result is consistent with those shown in Fig. 2.

The inter-regional synchronisations $F{{\rm{I}}{\rm{N}}}_{b}^{l,m}$ were highest in the eastern regions. All of them were greater than 0.7, especially the following four regional combinations that had very high degrees of synchrony: $F{{\rm{I}}{\rm{N}}}_{b}^{A,B}=0.844$, $F{{\rm{I}}{\rm{N}}}_{b}^{AC}\,=0.857,\,F{{\rm{I}}{\rm{N}}}_{b}^{B,C}=0.831,\,{\rm{a}}{\rm{n}}{\rm{d}}\,F{{\rm{I}}{\rm{N}}}_{b}^{B,D}=0.84$. In the western regions, only Shikoku’s inner-regional synchronisation was greater than 0.7 $(F{{\rm{I}}{\rm{N}}}_{w}^{G}=0.758)$. The inter-regional synchronisations between all pairs in the western regions (E–H) were less than 0.7.

The eight regional averages of in-phase matrices are illustrated in Fig. 4(c). Regional synchronisations in the eastern regions (A–D) were very strong, while those in the western regions (E–H) were less synchronised. A comparison of Figs 3(c) and 4(c) indicated that the in-phase and out-of-phase analysis was more appropriate than a correlation analysis for the two-cycle-based fluctuations.

**Figure 5:**
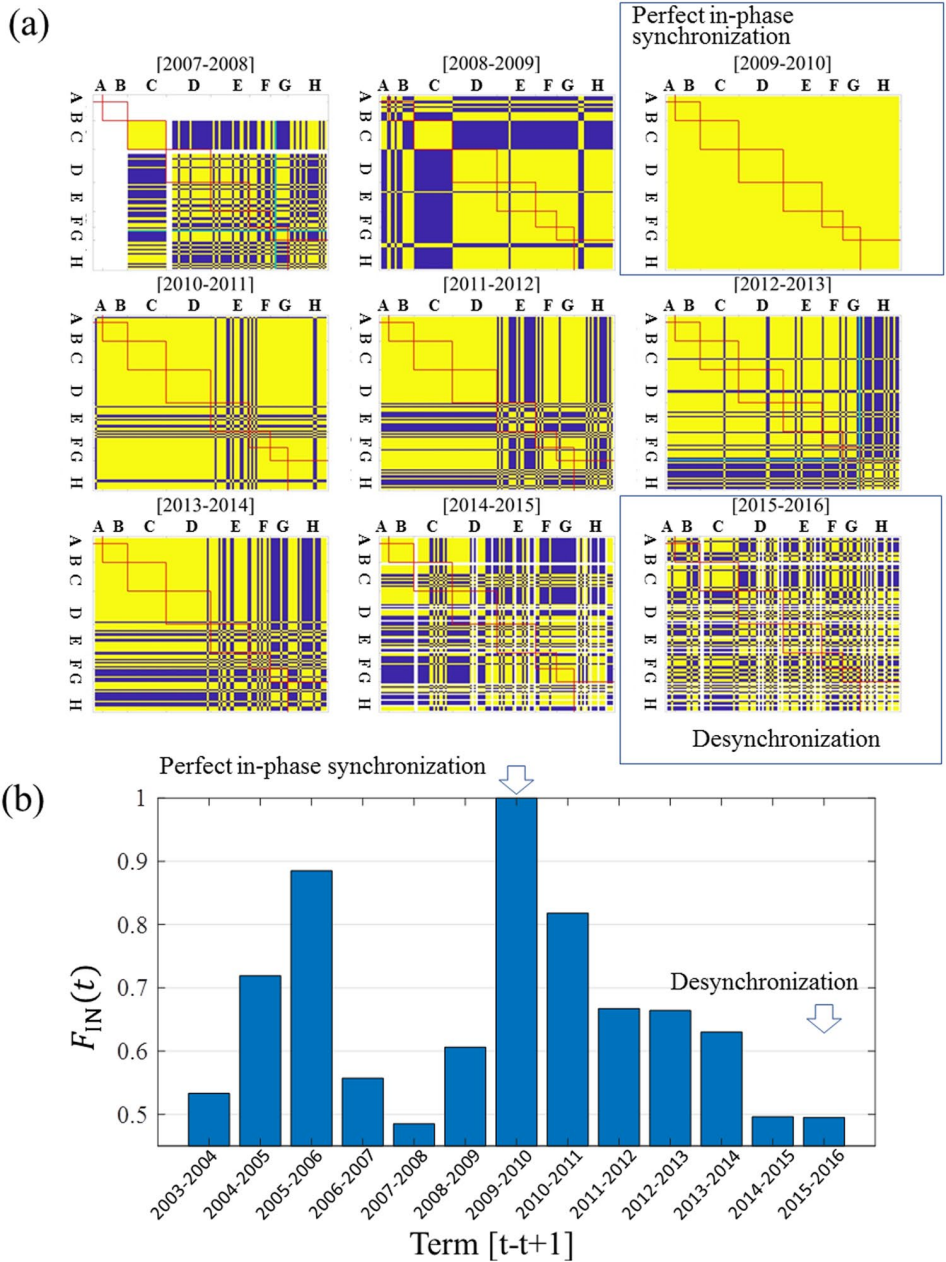
Intermittent nature of the synchronisations. (**a**) Time evolutions of in-phase matrix $[{f}_{{\rm{IN}}}^{i,j}(t)]$ developed from 2007–2008 through 2015–2016. Yellow cells indicate ${f}_{{\rm{IN}}}^{i,j}(t)=1$ (in-phase), and blue cells indicate ${f}_{{\rm{IN}}}^{i,j}(t)=0$ (out-of-phase). Green cells indicate sites in which the amount of pollen in a certain year was the same as that in the previous year (only two sites each in 2007–2008 and 2012–2013). White cells indicate a lack of data. (A) Hokkaido, (B) Tohoku, (C) Kanto, (D) Chubu, (E) Kansai, (F) Chugoku, (G) Shikoku, and (H) Kyushu. (**b**) Time evolution of the degree of in-phase synchronisation *F*_IN_(*t*) throughout Japan in each term [*t* − *t* + 1].

To show the time evolution of spatial synchrony, Fig. 5(a) exhibits snapshots of the matrix $[{f}_{{\rm{IN}}}^{i,j}(t)]$ for each [*t* *−* *t* + 1] term. Yellow cells indicate ${f}_{{\rm{IN}}}^{i,j}(t)=1$ (in-phase), and blue cells indicate ${f}_{{\rm{IN}}}^{i,j}(t)=0$ (out-of-phase). In the matrix for 2009–2010, all ${f}_{{\rm{IN}}}^{i,j}(2009)\,$ = 1, revealing that perfect in-phase synchronisation occurred throughout the 120 sites. It is a dramatic phenomenon that all 120 sites behaved in-phase throughout Japan. In 2008–2009, Kanto showed a very clear out-of-phase synchronisation with other regions. Interestingly, the matrix in 2015–2016 showed significant mixing of in-phase and out-of-phase synchronisation patterns, both inner- and inter-regionally. This result indicates that there was no significant phase synchronisation throughout Japan in 2015–2016. Furthermore, we can say that desynchronisation occurred in 2015–2016. From 2008 to 2016, in the eastern regions (A–D), both inner- and inter-regional synchronisation patterns have been strong. The only unique exception is Kanto, which had a very clear out-of-phase synchronisation with each of five other regions (D–H) in the 2008–2009 term. In the western regions, out-of-phase synchronisations (blue cells) occurred frequently between sites. With the snapshots displayed in Fig. 5(a), we were able to visualise the phase states of 120 × 120 site combinations per year.

Figure 5(b) shows clear intermittent synchronisation patterns in Japan over 14 years starting in 2003. *F*_IN_(*t*) denotes the degree of in-phase synchronisation throughout Japan in the [*t* − *t* + 1] term. ${F}_{{\rm{IN}}}(2009)=1.0$ indicated that perfect in-phase synchronisation occurred in 2009–2010. It is remarkable that perfect synchronisation occurred over the site to site distance of 1,613 km between sites 3 and 120. The three measurement periods, 2007–2008, 2014–2015, and 2015–2016, ${F}_{{\rm{IN}}}(t)\,{\rm{values}}$ were very close to 0.5. We noted that in Fig. 5(a), there were some significant out-of-phase clusters in 2007–2008 and 2014–2015 but not in 2015–2016. Therefore, we may say that almost perfect desynchronisation occurred in 2015–2016. Consequently, we determined that a perfect in-phase and an almost perfect desynchronisation occurred throughout Japan in 2009–2010 and 2015–2016, respectively.

### Concluding remarks

Using an in-phase and out-of-phase analysis, we found two important features of nationwide synchronisations of allergenic pollen coming mainly from sugi (*C. japonica*) and hinoki (*C. obtusa*) in Japan.

First, there was the difference in the degree of phase synchronisations between eastern and western Japan. In the four eastern regions, both inner- and inter-regional synchronisations were significant. In the four western regions, no significant synchronisation was identified.

Second, the nationwide synchronisation pattern was “intermittent synchronisation”, because there were yearly changes in the degree of in-phase synchronisation. Besides the perfect in-phase synchronisation in 2009–2010 and the almost perfect desynchronisation in 2015–2016, the other measurement periods demonstrated mixed in-phase and out-of-phase synchronisations, reflecting chimera-like states^29,30^.

The mechanism causing regional differences in the degree of synchronisation and nationwide intermittent synchronisation is still unknown. However, based on masting and/or alternate bearing, we hypothesise that the pollen synchronisation patterns are spatially distributed collective dynamics driven by pollen coupling and the Moran effect^27^. To clarify their causes, a comparison of the data and collective dynamics models should be conducted using an in-phase and out-of-phase analysis.
